# Supplementary figures and images for: Transient vascular occlusions in a zebrafish model of mycobacterial brain infection
Source: PLoS One. 2025 Sep 12;20(9):e0332161. doi: 10.1371/journal.pone.0332161 (PMC12431238; doi:10.1371/journal.pone.0332161)

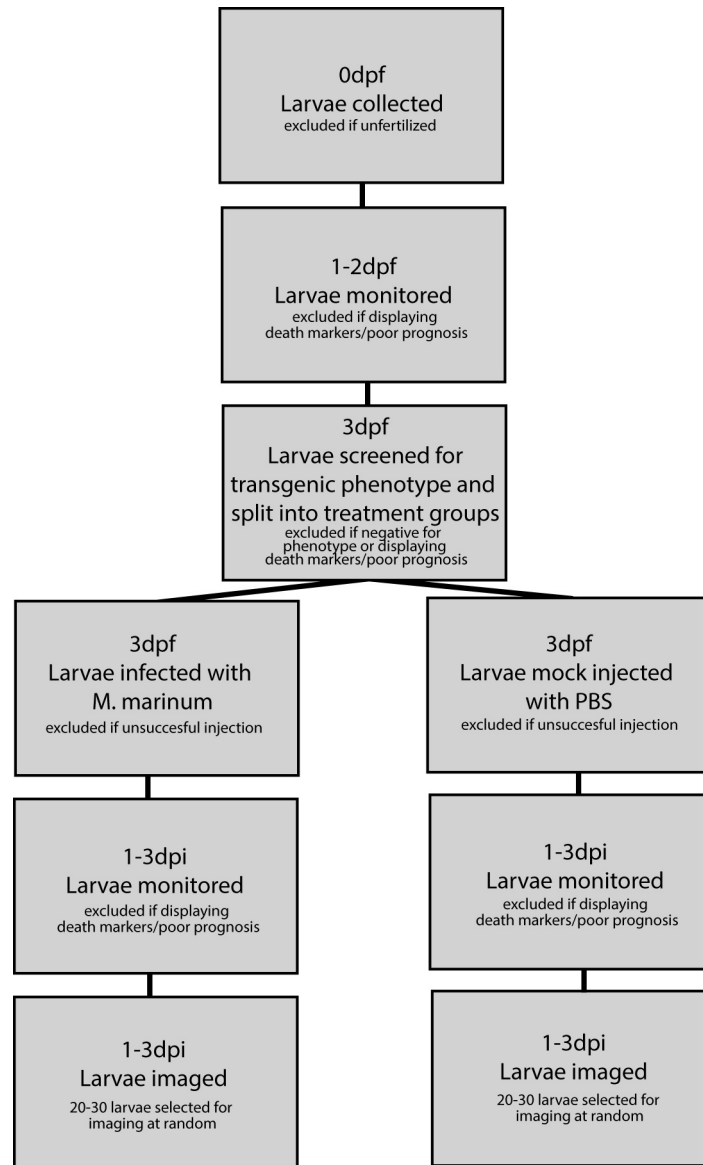

Supplement: S1 Fig — At 3 dpf, healthy transgenic larvae are infected in the caudal vein with M. marinum or injected with PBS. At 3 dpi, confocal imaging of larvae is used to assess infection and vessel changes in the brain. (PDF) [file pone.0332161.s001.pdf]
